# The impact of Stress Management and Resilience Training (SMART) on academic physicians during the implementation of a new Health Information System: An exploratory randomized controlled trial

**DOI:** 10.1371/journal.pone.0267240

**Published:** 2022-04-22

**Authors:** Edward G. Spilg, Hanna Kuk, Lesley Ananny, Kylie McNeill, Vicki LeBlanc, Brent A. Bauer, Amit Sood, Philip S. Wells

**Affiliations:** 1 Department of Medicine, University of Ottawa, Ottawa, Ontario, Canada; 2 Ottawa Hospital Research Institute, Ottawa, Ontario, Canada; 3 Department of Innovation in Medical Education, University of Ottawa, Ottawa, Ontario, Canada; 4 Department of Internal Medicine, Mayo Clinic, Rochester, Minnesota, United States of America; Osaka University Graduate School of Medicine, JAPAN

## Abstract

**Objective:**

The Stress Management and Resilience Training (SMART) program is an evidence-based intervention designed to build resilience in physicians in clinical practice. The objective of the current study was to assess the impact of the SMART program on academic physicians’ levels of resilience, subjective happiness, stress, and anxiety, and specifically during the implementation of a new hospital-wide Health Information System (HIS).

**Methods:**

A total of 40 physicians in a tertiary care academic hospital were randomized (allocation ratio 1:1) to either the SMART intervention or the control condition. The SMART intervention consisted of one mandatory two-hour in-person workshop and an optional 24-week online program, designed to support the materials delivered in the workshop. Outcome measures were assessed using validated scales administered online at baseline and at 3-months and 6-months follow-up.

**Results:**

After adjusting for baseline levels of each outcome, no statistically significant intervention effect was observed for resilience, subjective happiness, stress or anxiety at 3-months or 6-months follow-up. However, physicians in the intervention group demonstrated improvements in resilience, stress and anxiety at follow-up that were within the range of clinically relevant differences.

**Conclusions:**

The findings of this exploratory study provide modest support that the SMART intervention may be beneficial for proactively addressing physician wellness during the implementation of a new HIS and that larger randomized trials are warranted.

**Trial registration:**

NCT04384861.

## Introduction

Operational challenges are increasingly stressful in physicians’ clinical practices. One such operational challenge faced by physicians is their interaction with the Health Information System (HIS). The HIS is associated with less time spent face-to-face with patients [1] and increased time on patient-related administrative tasks often during personal time in the evenings [2]. Prior to implementation of HISs, physicians report a number of perceived barriers to acceptance, in particular relating to the organizational structures and change management processes [3]. Following implementation, HIS design and use have both been associated with clinician stress and burnout [4].

Evidence supports the use of interventions both at an organizational and individual level to decrease physician burnout [5]. For instance, a recent systematic review identified that a number of organizational-directed workplace interventions have been shown to lessen physician burnout [6]. With respect to the HIS, however, with the exception of incorporating scribes or medical assistants to complete HIS documentation and tasks, there is evidence only from poor quality studies suggesting that HIS training and technical improvements of HISs reduce burnout [6]. There is also evidence, albeit of lower strength, to support the use of resilience-building interventions to mitigate against the effects of burnout, stress and anxiety in physicians [7, 8]. Resilience reflects the personal qualities that enable individuals to thrive when facing adversity [9]. Given its adaptive role in stress-coping, developing resilience may enable physicians to better cope with organizational stressors, such as a novel HIS, and in turn, help reduce the degree to which they appraise situations in their lives as stressful [10]. However, there is limited evidence to support the use of such interventions both before and during HIS implementation.

The Stress Management and Resilience Training (SMART) program is one of the few evidence-based interventions designed to build resilience in physicians in clinical practice [7, 8]. Developed at the Mayo Clinic and originally adapted from Attention and Interpretation Therapy, the SMART program is a brief intervention, typically delivered in a single 90-minute workshop [11, 12]. The structured program teaches physicians attentional training through daily practices to help reframe potentially stressful situations more quickly and decrease the impact of stress on the brain [13]. In turn, these practices help clinicians recognize different modes of thinking of the brain and cultivate a more flexible disposition in order to reconnect with the meaning of their work and their relationships [13]. Randomized pilot trials using the SMART program in the United States have shown improvements in resilience and decreases in stress, anxiety and burnout in physicians [11, 12]. Although the SMART program has yet to be evaluated in the context of a new HIS, its brief format is well-suited to periods of transition where physicians face additional operational stressors.

On June 1, 2019, The Ottawa Hospital (TOH) adopted a new HIS, Epic (Epic Systems Corporation, Madison, Wisconsin). In light of the lack of evidence-based interventions to support physicians facing this operational stressor, we conducted an exploratory RCT of the SMART program concurrently with the launch of Epic. The objective of the current study was to assess the impact of the SMART intervention program on academic physicians’ levels of resilience, subjective happiness, stress, and anxiety during the implementation of a new hospital-wide HIS which was being implemented concurrently with the study in order to inform future studies.

## Methods

### Study design

Faculty physicians in the Department of Medicine at a large academic tertiary care hospital in Canada were randomized (allocation ratio 1:1) in a parallel-group controlled trial in 2018–2019. The trial was conducted evaluating the impact of the SMART program as compared to a control group. The study took place concurrently with the implementation of the hospital’s new HIS, Epic. Specifically, Epic went live in June 2019, with the main physician system training taking place March-April 2019. Thus, during data collection (January-August 2019), the launch of the new HIS system was the major institutional stressor facing physicians.

We obtained ethics approval from the Ottawa Health Science Network–Ottawa Hospital Research Ethics Board (OHSN-OHREB; Protocol ID 20180536-01H). The SMART trial was registered at the US National Institutes of Health (ClinicalTrials.gov) #NCT04384861 (https://clinicaltrials.gov/ct2/show/NCT04384861). Due to an administrative error, the trial was registered retrospectively on May 12, 2020. The trial protocol and CONSORT checklist are available as S1 Checklist.

### Study population

All 261 full-time faculty physicians in the hospital’s Department of Medicine were invited to participate in the study via email. Eligibility criteria included having a full-time academic appointment in the Department. No exclusion criteria were delineated. Physicians were enrolled in a first-come, first-served basis between August and November 2018. Participation and study enrolment were voluntary. Written informed consent was obtained for all physicians.

Based on a power calculation using data from previous pilot studies [11, 12], we set the recruitment target at 40 physicians (20 in the Active group, 20 in the Control group). Specifically, we estimated that 40 physicians (20 per group) would be needed to detect a difference of 1 standard deviation between the intervention and control groups on the continuous outcome variables (power of 0.85; *p* < 0.05) for this exploratory study.

#### Randomization

After study enrolment, an independent researcher (LA), who was not involved in conducting the intervention, randomly assigned physicians to the intervention (Active group, n = 20) or the control condition (Control group, n = 20; allocation ratio 1:1) using the random number generator function in MS Excel. Both recruitment and randomization procedures were carried out by the same researcher and randomization was concealed from the other study team members. Blinding was not applicable to the current study as both the research team and physicians were aware who received the intervention.

### Study intervention

Physicians assigned to the Active group received the SMART program; those assigned to the Control group received no intervention. The SMART program is a brief, evidence-based, intervention developed by a leading expert in stress and resiliency (AS). Guided by the principles of gratitude, compassion, acceptance, forgiveness and higher meaning, individuals learn self-care practices to enhance their resiliency and reduce their emotional and physical vulnerability to daily stressors [11]. A more detailed description of the SMART program is available elsewhere [12, 13].

The SMART intervention consisted of one mandatory two-hour in-person workshop and an optional 24-week online program from the Mayo Clinic, developed to support the materials delivered in the workshop. While previous SMART programs have included refresher sessions usually by regular teleconferences involving workshop participants [12, 13], this was the first time an online program was used to support the content delivered in the workshop. The workshop was delivered by a physician and trained SMART program facilitator (ES). The content of the workshop was standardized and accompanied by a PowerPoint presentation and delivered in an interactive format to allow for group discussions. To accommodate physicians’ schedules, physicians attended one of six identical workshops that were held at different times and locations on the hospital’s 3 campuses in late January and early February 2019.

The optional online program began one week after the workshop and consisted of two portions: the 4-week TRAIN (4 modules of approximately 45 minutes each, 1 per week; January-March 2019) and the 20-week SUSTAIN portion (20 modules of approximately 10–15 minutes, 1 per week; February-August 2019). The online program was hosted by the Mayo Clinic and the research team did not have access to the data. Of note, the intervention was designed to last 24 weeks; however, physicians had access to the online modules up until they were discontinued by the Mayo Clinic in November 2019.

### Study outcomes

The outcomes of the study were physicians’ self-reported levels of a) resilience, b) subjective happiness, c) stress, and d) anxiety, which were assessed using four validated scales (described below) administered electronically via an online survey. Using a parallel design, baseline data on all four outcomes were collected from physicians in both the Active and Control groups prior to the intervention (January 2019). Demographic data (i.e., age, sex, division, year of graduation from medical school and year of completion of residency training) were also collected. Study outcomes were assessed again at 3-months (April-May 2019) and at 6-months (July-August 2019) follow up. Due to an administrative error, data were not collected at 1-month follow-up, as scheduled in the protocol. Physicians in the Active group were invited to attend 1 of 3 focus groups in May/June 2019 to discuss their experiences with the study. Results arising from these focus groups will be published in a separate manuscript.

### Outcome measures

#### Resilience

The Connor–Davidson Resilience Scale (CD-RISC) was utilized to assess self-reported levels of resilience and the ability to cope with adversity [9]. Physicians provided their responses on a 25-item scale ranging from 0 (not true at all) to 4 (true nearly all the time) and the scores were summed for each participant with the possible score range from 0 to 100. Higher scores are indicative of greater levels of resilience [9].

#### Subjective happiness

The 4-item Subjective Happiness Scale (SHS) was used to assess physicians’ levels of global subjective happiness [14]. Physicians were asked to characterize themselves using an absolute happiness rating as well as happiness ratings in relation to their peers and happy and unhappy individuals in general. Scoring was reversed for the last item and a single composite score was obtained by averaging the scores on all 4 items, with higher scores reflecting greater happiness [14].

#### Stress

The 10-item Perceived Stress Scale (PSS) is a long-standing, standardized stress assessment instrument comprised of 10 items scored on a 5-point Likert scale ranging from “never” (0) to “very often” (4) [10]. Six of the items are negative (e.g., “In the last month, how often have you felt nervous and “stressed”?) and the remaining 4 items are positive (e.g., “In the last month, how often have you felt that you were on top of things?”). To obtain a global level of perceived stress, the 4 positive items were reversed scored and all items were summed (range from 0 to 40, with higher scores indicative of higher levels of stress) [10]. Cut-off scores from previous studies were used to classify low (0–13), moderate (14–26), and high (27–40) stress in our sample [15–17].

#### Anxiety

The Generalized Anxiety Disorder-7 item (GAD-7) scale was utilized to assess the severity of physicians’ self-reported anxiety symptoms [18, 19]. The scale is a screening tool comprised of seven items which are scored from 0 to 3, with a maximum score of 21. Cut-off scores of 5, 10, and 15 delineate mild, moderate, and severe anxiety, respectively, while scores ≤4 correspond to minimal anxiety [19].

### Statistical analysis

All data were analyzed using SPSS v28.0. Analyses were performed using data from physicians who completed measures at baseline and at 3-month follow-up. Descriptive statistics were calculated to describe the sample characteristics at baseline. All outcome measure data were inspected for normality using the Kolmogorov-Smirnov test and visual inspection of histograms. For data that deviated from normality, we performed a log_10_ transformation with the addition of 1 as a constant to account for zeros in our data. These data are reported as medians (md) and interquartile ranges (IQR). Normally distributed data are presented as means (M) and standard deviations (SD). The intervention effect was measured by an analysis of covariance (ANCOVA) to control for baseline values. Thus, for each outcome, the measure at 3-months and 6-months were the dependent variables, with the baseline measure as the covariate and group (Active or Control) as the independent variable. The ANCOVA models were conducted separately for each follow-up timepoint. Preliminary checks were performed to ensure there were no violation of assumptions, including linearity, homogeneity of variances, and homogeneity of regression slopes. The least mean square differences (LSMD) between the two groups and corresponding 95% confidence intervals (CI) are reported for each outcome. We computed partial eta squared (η_*p*_^2^) to determine effect size, with 0.01, 0.06, and 0.138 corresponding to small, medium and large effects, respectively [20]. A two-tailed significance level was set at *p* < 0.05, with no adjustment for multiple comparisons given the exploratory nature of this study.

## Results

### Study population

Physician characteristics and demographics data are provided in greater detail in Table 1. Thirteen (13) physicians in the Active group were male and 7 physicians were female, with a mean age distribution of 45.9 ± 9.1 years and 14.0 ± 9.7 years in independent practice. In the Control group, 11 physicians were male and 7 physicians were female, with demographic information not voluntarily provided by 2 physicians. Similar to the Active group, the mean age distribution of the Control cohort was 45.9 ± 10.1 years, with an average of 15.6 ± 9.6 years in independent practice.

**Table 1:** Demographic characteristics and participation rates for physicians in the Active and Control groups.

Variable		Active (N = 20)		Control (N = 20^1^)	
		N (%)	Mean±SD	N (%)	Mean±SD
Sex	Male	13 (65%)		11 (55%)	
	Female	7 (35%)		7 (35%)	
Age			45.9±9.1		45.9±10.1
Years in independent practice			14.0±9.7		15.6±9.6
Division	General Internal Medicine	4		4	
	Hematology	2		3	
	Palliative care	2		2	
	Neurology	2		2	
	Cardiology	3		2	
	Geriatrics	3		1	
	Endocrinology	1		1	
	Physical Medicine & Rehabilitation	2		--	
	Respirology	--		1	
	Nuclear Medicine	1		--	
	Epidemiology	--		1	
	Infectious Diseases	--		1	
Participation Rate	Baseline	20 (100%)		19 (95%)	
	3-month follow-up	19 (95%)		17 (85%)	
	6-month follow-up	16 (80%)		16 (80%)	

^1^Demographic information was not provided by 2 physicians in the Control group.

All 20 physicians in the Active group participated in the SMART program workshop; 17 physicians accessed the optional online TRAIN modules and 13 of those physicians accessed the SUSTAIN modules after completion of the TRAIN portion.

**Fig 1:**
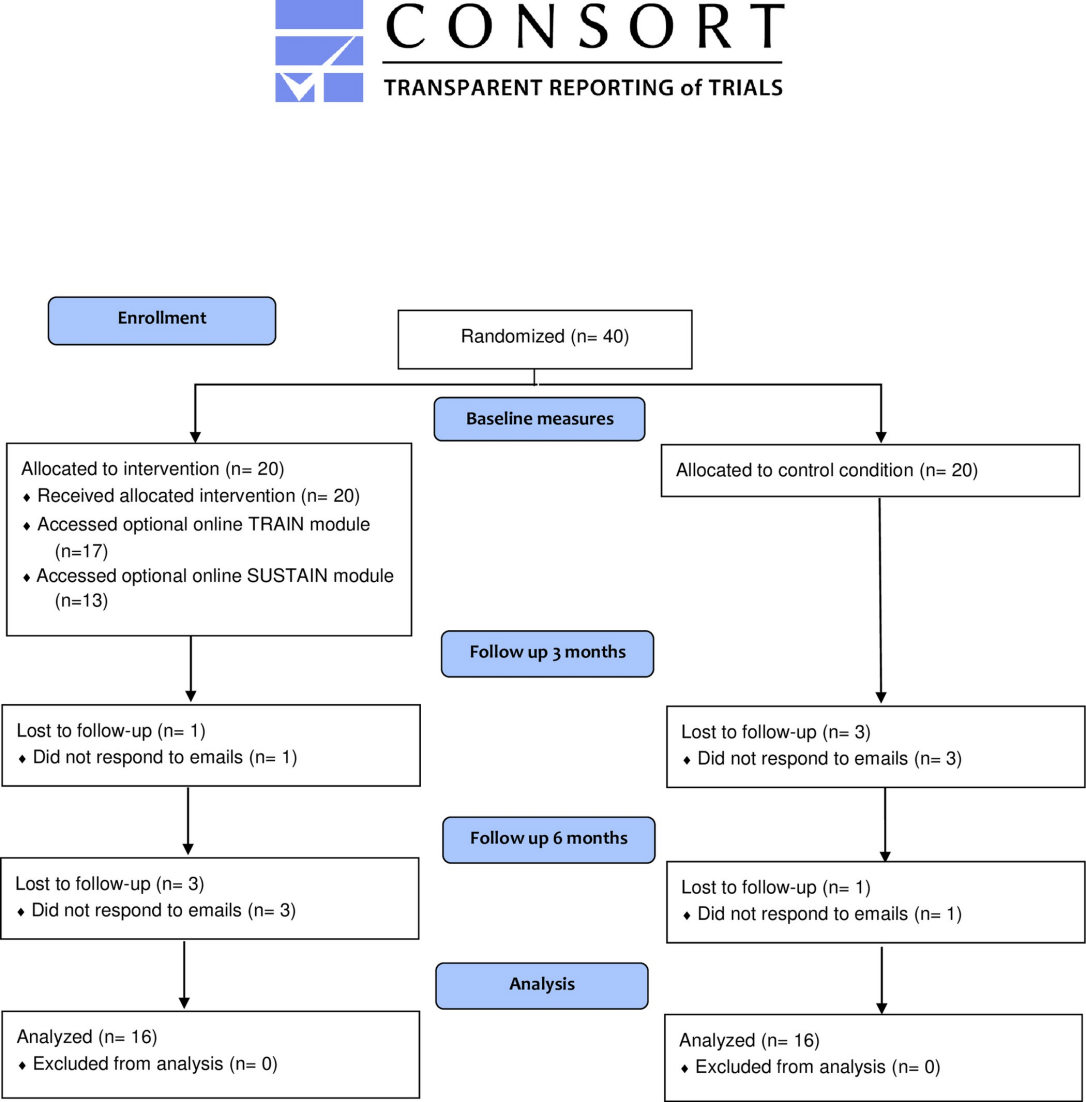
Flow chart of participants. Follow-up and drop-out of participants in the Active and Control groups.

Nineteen (19) physicians in the Active group and 17 in the Control group provided complete data at baseline and at 3-month follow-up and thus continued with the study (Active: 95% and Control: 85% participation rate; Table 1). The 4 physicians who did not provide data at 3-months follow-up were removed from the analyses since the main outcomes could not be assessed. Three (3) additional physicians in the Active group and 1 participant farom the Control group were subsequently lost to the final follow-up at 6-months (Active n = 16; Control n = 16; Table 1; Fig 1).

### Outcomes

Upon inspection of the histograms, the GAD-7 data were positively skewed at all three timepoints and the Kolmogorov-Smirnov tests were statistically significant at baseline (*p* = 0.003) and 3-months follow-up (*p* = 0.027). Therefore, we calculated all GAD-7 data using log transformed data. The remaining outcome measures were all normally distributed.

Summary statistics for the outcome measures at baseline, 3-months follow-up and 6-months follow-up in Table 2. The raw mean reported levels of the PSS at baseline met the classification criteria of “moderate stress” in both the Active and Control groups [16, 17]. This classification was also met at 3- and 6-months follow-up for both groups. Similar outcomes were observed for the GAD-7 median scores, as the definition of “mild anxiety” was met in the Active group at baseline and at 3- and 6-months follow-up. The Control group reported “minimal anxiety” at baseline and “mild anxiety” at 3- and 6-months follow-up [19].

**Table 2:** Summary statistics for Active and Control group on study outcome measures.

		Active			Control		
Outcome		Baseline (N = 20)	3-months follow-up (N = 19)	6-months follow-up (N = 16)	Baseline (N = 19)	3-months follow-up (N = 17)	6-months follow-up (N = 16)
Resilience (CD-RISC), M (SD)		69.35 (7.71)	73.21 (9.05)	73.69 (10.90)	68.42 (11.82)	66.59 (10.10)	66.88 (10.01)
Subjective Happiness (SHS), M (SD)		5.24 (0.93)	5.34 (1.04)	5.25 (1.22)	5.21 (0.98)	5.13 (0.92)	5.09 (1.01)
Stress (PSS), M (SD)		17.75 (5.48)	15.21 (6.61)	14.88 (8.16)	15.74 (4.02)	16.82 (5.39)	17.75 (7.81)
Anxiety (GAD-7), Md (IQR)		5.00 (4.00, 6.00)	6.00 (2.40, 7.45)	5.48 (2.00, 9.53)	4.00 (2.00, 6.00)	5.00 (3.00, 8.75)	6.32 (2.21, 10.72)

In the absence of available cut-off scores for the CD-RISC or the SHS, the mean levels of resilience for both groups at baseline are lower than those reported by a historical cohort of physicians in our Department of Medicine [21], corresponding to below the 25^th^ percentile for the general US population [9]. These levels were maintained in the Control group at 3- and 6-months follow-up, while those reported by the Active group are comparable to 8-week post-intervention scores reported by physicians who participated in a previous SMART intervention [11]. The mean levels of the SHS in the Active and Control groups were higher than those reported in previous studies with physicians [22–24] at baseline and 3- and 6-months follow-up.

Results of the ANCOVAs are presented in Table 3. After adjusting for baseline levels of each outcome, no statistically significant differences were observed between the Active and Control group for any of the four outcomes at 3-months and 6-months follow-up. An examination of the LSMDs, however, shows a pattern of improvement in favour of the intervention at both 3- and 6-months follow-up for the CD-RISC, PSS and GAD-7. Although the differences between the two groups were not statistically significant, medium effect sizes were reported for the CD-RISC (η_*p*_^2^ = 0.10) and GAD-7 (η_*p*_^2^ = 0.057) at 3-months follow-up. Medium effect sizes were also reported at 6-months follow-up for the CD-RISC (η_*p*_^2^ = 0.096), PSS (η_*p*_^2^ = 0.085) and GAD-7 (η_*p*_^2^ = 0.111). The remaining effects sizes were small.

**Table 3:** Results of the ANCOVAs and least square mean differences for study outcome measures at 3-months and 6-months follow-up.

	3-month follow-up (N = 36)				6-months follow-up (N = 32)			
Outcome	LSMD	95% CI	*p*-value	η_*p*_^2^	LSMD	95% CI	*p*-value	η_*p*_^2^
Resilience (CD-RISC)	5.35	-0.21, 10.91	0.059	0.104	4.85	-0.81, 10.05	0.090	0.096
Subjective Happiness (SHS)	0.14	-0.28, 0.56	0.508	0.013	0.23	-0.23, 0.69	0.316	0.035
Stress (PSS)	-2.45	-6.38, 1.49	0.214	0.046	-4.83	-10.85, 1.19	0.111	0.085
Anxiety (GAD-7)								
Log-Scale	-0.14	-0.35, 0.06	0.167	0.057	-0.25	-0.52, 0.02	0.068	0.111
Relative Difference^1^	0.72	0.45, 1.15	--	--	0.56	0.30, 1.05	--	--

^1^exp(log difference) giving % difference in medians.

There was a strong relationship between scores at baseline and 3-months follow-up for the CD-RISC (*p* < 0.001), SHS (*p* < 0.001) and the GAD-7 (*p* < 0.001) with large effect sizes (η_*p*_^2^ = 0.299, 0.61 and 0.289, respectively). This relationship was also observed at 6-months follow-up for the CD-RISC (*p* < 0.001), SHS (*p* < 0.001) and the GAD-7 (*p* = 0.002) with large effect sizes (η_*p*_^2^ = 0.471, 0.68 and 0.275, respectively). The covariate was not statistically significant in the ANCOVAs for the PSS at 3-month (p = 0.078) or 6-month (p = 0.086) follow-up (data not shown).

### Program-related adverse events

No harms or unintended effects in either the Active or Control groups were observed.

## Discussion

The objective of the current exploratory study was to assess the impact of the SMART intervention program on academic physicians’ levels of resilience, subjective happiness, stress, and anxiety, and specifically during the implementation of a new hospital-wide HIS which was implemented concurrently with the study. To our knowledge, this is the first physician wellness intervention implemented during the context of an HIS organizational change. It is also the first time the SMART program has been evaluated in the Canadian healthcare context and that an online program has been used to reinforce and sustain workshop participant learning and ongoing reflective practice. We found no statistically significant intervention effect on physicians’ levels of resilience, subjective happiness, stress or anxiety compared to the control condition at 3- or 6-months follow-up, after controlling for baseline levels of these outcomes. Despite trying to ascertain appropriate statistical power with our power calculation, our analyses were underpowered, given 20% attrition in both study arms.

Although we did not detect a statistically significant difference between groups, physicians in the active group did show improvements in resilience, stress and anxiety at both follow-up timepoints, which merits discussion. Interpretation of the outcomes is not straightforward, given the lack of published minimal clinically important differences (MCID) for these outcome measures, which reflects the smallest difference in scores that is considered to be meaningful or clinically important [25]. In previous studies, the average MCID for the GAD-7 has been estimated at between 1.5 and 4 [25–27], though for “mild” baseline levels, a MCID of 1 has been put forth [26]. Moreover, the research collectively defines a MCID as an improvement of 20–30% in GAD-7 scores [26, 27]. In our study, the relative reduction in median scores at 3-months was 28% (95% CI = 55% relative reduction to 15% relative increase) and at 6-months was 44% (95% CI = 70% relative reduction to 5% relative increase), thus well within the range of a clinically significant improvement. Less has been published on the MCID for the PSS; however, a range of values between 2.19 and 11 [16, 28, 29] has been estimated in the literature. The LSMDs at 3-months (-2.45, 95% CI = -6.38–1.49) and at 6-months follow-up (-4.83, 95% CI = -10.85–1.19) fall within the lower end of this range, and are greater than the MCIDs reported in a study of medical students (2.19–2.66) [16].

We are unaware of any published MCID for the CD-RISC. The observed LSMDs for resilience at both time-points represent greater than 0.5 standard deviation change from baseline in the Active group, which has been identified as the threshold for meaningful change in health-related quality of life measures [30], but is less than the 1 standard deviation change reported by physicians in a previous SMART study [11]. Based on the LSMD for the SHS, subjective happiness did not respond meaningfully to the intervention at either time-point; however, our study may have been subject to a ceiling effect, given the high mean levels on the SHS reported by the sample at baseline. It is also possible that subjective happiness is less amenable to the intervention due to the various contextual and personality factors underlying this construct [31]. Future research is needed to elucidate the nature of the association between subjective happiness and symptomatic outcomes (i.e., anxiety and stress).

Overall, these exploratory findings are promising and can be used to inform larger randomized controlled trials of the SMART program; specifically, in terms of determining the sample size needed for future trials. Future research is also needed to determine the MCID for these outcome measures in order to better interpret the effectiveness of the SMART and other resilience-based interventions. From an organizational perspective, understanding what a clinically meaningful change is may be more important than detecting a statistically significant change in outcomes for wellness interventions to make an impact in the real world.

These findings also contribute to data regarding the effectiveness of the SMART program for physicians. In previous pilot studies of brief SMART programs with physicians, Sood and colleagues reported a reduction of stress and anxiety at 8 weeks [11] and 12-weeks [12] follow-up, with large effect sizes. Results for the intervention effect on resilience were mixed as Sood and colleagues did not find a statistically significant difference in resilience for radiologists in the intervention group [12], yet Sood and colleagues reported a statistically significant improvement in resilience, with a large effect size, in their sample of academic physicians [11]. In contrast to these studies, we controlled for baseline scores in our analyses, which were found to be highly correlated with resilience, happiness and anxiety measured at both 3- and 6-months follow-up. Physicians in both study groups also reported low levels of stress and anxiety and high levels of happiness at baseline, suggesting this sample was relatively psychologically healthy, with marginal ability to improve these outcomes in the sample. In contrast, physicians in the previous SMART studies reported higher levels of stress and anxiety at baseline [11, 12]. Future trials should consider baseline measurements of these outcomes in terms of establishing inclusion criteria and accounting for heterogeneity in these outcomes.

Furthermore, in the aforementioned study of the SMART program with academic physicians, Sood and colleagues’ intervention involved a one-on-one workshop [11] while other SMART studies have offered optional one-on-one follow-ups [13]. Our intervention involved a group workshop with an optional online support program. It is possible that more individualized on-going support (e.g., follow-up phone calls, refresher sessions) may be required for the intervention to be effective [13]. Our study also included a longer follow-up period of 6-months to assess the long-term effects of the SMART program [8, 11]. Although not tested statistically, we observed that the effect of the intervention increased over time for stress and anxiety as the LSMDs were greater and *p*-values were lower at 6-months follow-up. Between the 3- and 6-month data collection periods, 11 physicians in the active group participated in focus groups to discuss their experiences with the study. It is possible that these group discussions acted as a form of a refresher course that prompted a greater reduction in stress and anxiety at the end of the study. Moreover, this study marked the first time an optional online program had been used to support to the SMART intervention, with materials available throughout the 6-month study period in order to reinforce the content of the workshop. It is possible that there was a continuation effect with greater exposure to intervention content over time, as 85% of the Active group accessed the first part of the online program, and 65% accessed the second portion.

A novel aspect of this study was the adoption of a new hospital-wide HIS which went live at TOH between the 3- and 6-month follow-up. It has been noted previously that some of the metrics that predict a successful HIS implementation, including stakeholder engagement, commitment and involvement [32, 33], may be related to factors that contribute to stress, anxiety, and burnout in physicians, such as the lack of input or control regarding issues affecting their work lives [34]. Resilience reflects one’s capacity to respond and adapt to challenging situations [9] and may bear particular relevance for physicians undergoing organizational transitions, as personal competencies related to tolerance of stress and negative influences may not be emphasized in medical training [35]. Indeed, studies have found that greater intolerance of ambiguity and uncertainly is predictive of higher levels of perceived work-related stress and burnout in physicians [36, 37]. From an organizational perspective, the value of studying the SMART program during major organizational change is that this intervention represents a tool that can be used strategically when organizational change is anticipated to increase stress, whether is it internal and planned (e.g., a new HIS) or external and unplanned (e.g., a pandemic). Although we did not find a statistically significant intervention effect for any of our outcomes at 3- or 6-month follow-up in this exploratory study, the findings offer promising preliminary data that further research is warranted using a larger randomized trial to evaluate the effectiveness of the SMART program for physicians facing organizational stressors, such as the transition to a novel HIS.

### Strengths and limitations

In terms of strengths, we employed a randomized design and assessed outcomes at 6-months, a longer follow-up than in previous SMART programs [8]. We used validated scales to assess all study outcomes. The intervention was evidence-based and multi-modal, involving both a didactic workshop and an optional online support program. The content of the workshop and online program were standardized, and the quality of delivery was ensured by the accreditation of the workshop facilitator. The accessibility and short time-commitment of the intervention increases the scalability of the intervention, particularly in the context of organizational change (i.e., the implementation of a HIS). Our exploratory study also extends previous SMART studies as it was the first time that this intervention program was delivered outside of the group who developed the program originally [11–13] and in a Canadian healthcare context.

Study limitations include the relatively small sample size, self-selection bias and lack of active intervention in the control group. Although sufficient power was ascertained with our power calculation [11, 12], we did not account for study attrition and therefore our analyses were underpowered, increasing the risk of a Type II error. While the participation rate was high at 3-months, we observed a loss of 20% in both the Active and Control groups at the final timepoint and information on program dropout was not collected. This attrition rate is comparable to other resilience-based interventions [8] but higher than those reported in previous SMART interventions [11, 12]. It is possible that more one-on-one support helped boost retention in the intervention group in previous studies [11].

We employed a convenience sample of physicians who volunteered to participate in the study and thus may be subject to a self-selection bias. It is likely that enrolment included physicians who were highly motivated to learn resiliency skills. Indeed, we observed low baseline levels of stress and anxiety in our sample, which limits generalizability of our findings among those with poor psychological health. Furthermore, we did not employ stratified randomization to balance the groups on participant characteristics that may have influenced the outcomes, such as gender or years of work experience, therefore making it more difficult to generalize the findings [38]. Given the nature of our outcome variables, we relied exclusively on self-reported measures; however, we did not control for social desirability in the study. It should also be noted that the GAD-7 is a screening instrument and not a diagnostic tool for anxiety [19]. Further, since there is no standard of care for this situation, our control group received no active intervention. We thus cannot exclude the possibility that some of the intervention effect was related to expectancy and group participation. However, the group participation was limited to a single 2-hour session. Finally, we did not control for any other concomitant interventions that may have acted on the psychological health of the physicians in our study.

## Conclusions

Academic physicians who participated in the SMART program during the implementation of a new hospital-wide HIS demonstrated improvements in resilience, stress and anxiety that were within the range of clinically relevant differences; however, the results were not statistically significant after controlling for baseline scores on these outcomes. The findings of this exploratory study provide modest support that the SMART intervention may be beneficial for proactively addressing physician wellness during the implementation of a new HIS.

## Supporting information

S1 Checklist CONSORT checklist. (DOC)

S1 Protocol Trial protocol. (DOCX)
