# Correction: The impact of Stress Management and Resilience Training (SMART) on academic physicians during the implementation of a new Health Information System: An exploratory randomized controlled trial

**DOI:** 10.1371/journal.pone.0302288

**Published:** 2024-04-11

**Authors:** The PLOS ONE Staff

## Notice of republication

This article was republished on March 28, 2024, to correct an error in the XML version. A spelling mistake of the word "Resilience" in the title occurred during typesetting. The publisher apologizes for this error.Reference

## References

[1] Spilg EG, Kuk H, Ananny L, McNeill K, LeBlanc V, Bauer BA, et al. (2022) The impact of Stress Management and Resilience Training (SMART) on academic physicians during the implementation of a new Health Information System: An exploratory randomized controlled trial. PLoS ONE 17(4): e0267240. 10.1371/journal.pone.0267240. 35452478 PMC9032401

