# Supplementary material for: The impact of Stress Management and Resilience Training (SMART) on academic physicians during the implementation of a new Health Information System: An exploratory randomized controlled trial
Source: PLoS One. 2022 Apr 22;17(4):e0267240. doi: 10.1371/journal.pone.0267240 (PMC9032401; doi:10.1371/journal.pone.0267240)
Supplement: S1 Protocol — (DOCX) [file pone.0267240.s002.docx]

**Study title**

**Phase three: Improving physician empathy, compassionate care and wellness through the development of resilience-building communities of practice and creating a culture of empathy.**

**Background**

Definitions of resilience vary according to the context in which it is discussed. It is often considered from the perspective of the individual. Connor & Davidson, 2003,^1^ describe it as “the personal qualities that enable an individual to thrive in the face of adversity”. Over a life course, it is “the capacity and dynamic process of adaptively overcoming stress and adversity while maintaining normal psychological and physical functioning” (Wu et al., 2013)^2^ or “a dynamic capability which can allow people to thrive on challenges given appropriate social and personal contexts” (Howe at al., 2012)^3^. Social resilience is a term more commonly used in an ecological context and has been defined as the ability of a community to cope with and adapt to stresses such as social, political, environmental or economic change (Adger et al, 2000)^4^. Little has been written about social resilience as a construct within healthcare settings, however. It is postulated, however, that social resilience is closely related to the “culture” within an organization and that a model exploring communities of practice may be useful in trying to further understand the processes involved in culture change within such settings^5–7^.

Various studies have now shown a link between individual resilience and various mental health outcomes such as burnout, secondary traumatic stress, depression, and anxiety (Mak et al., 2011^8^; Mealer et al., 2012^9^; McGarry et al., 2013^10^; Lü et al., 2014^11^). In a systematic review by Fox et al., 2017^12^, 22 studies explicitly stated an aim of improving physician resilience. However, there was a lack of consensus concerning the conceptual understanding of resilience with low methodological rigour of the included studies. The authors recommended “methodologically rigorous research is required to establish best practice in improving resilience among physicians and to better consider how healthcare settings should be considered within interventions”.

The Mayo Clinic is considered one of the world’s leading centres in promoting physician wellness and reducing burnout. Dr. Amit Sood, Professor of Medicine, Mayo Clinic, Rochester, MN, has developed the Stress Management and Resilience Training (SMART) program, one of the very few evidence-based interventions which measured resilience in physicians (non-learners) using validated scales, and which found significant improvement in resilience^13,14^. The^1^ program, an educational 2-hour neuroscience-based intervention, is mandatory for all physicians, nurses and students across the Mayo Clinic Enterprise. Participants are trained in practices with the intention to help them develop intentional attention and the ability to reframe potentially stressful situations more quickly. The expectation is that this helps care providers reconnect to the meaning of their work and boosts engagement, which in turn influences their patient’s experiences.

**Research Questions**

1. What effect will an evidence-based resilience building intervention have on levels of resilience, stress and subjective happiness in Department of Medicine Faculty at the University of Ottawa?
2. How might implementation of an evidence-based resilience building intervention on Department of Medicine faculty, lead to the development of a community of practice for physician wellness in the Department of Medicine at The Ottawa Hospital/University of Ottawa? Also, will this further increase knowledge and understanding of the process through which behaviour (individual resilience) and culture (social resilience) change occurs within an academic healthcare organization?

**Recruitment and Procedures**

All academic physicians in the Department of Medicine, University of Ottawa will be invited via an email sent by the departmental administrator to attend a workshop on resilience. Participants will be able to express their interest in participating by clicking on a link in the invitation email. They will then be sent a link to our on-line consent form and first set of questionnaires. Participants will be asked to complete a written consent form for all phases of the study at the outset. Prior to signing the consent form, participants will have the opportunity to ask any questions they may have.

We aim to recruit 40 participants in total. Participants will be randomized into two groups of 20 physicians. Each participant will be given a number (1-40) and assigned to group A or group B using a random number generator (https://www.randomizer.org/). Participants in Group A will be the Active Arm and will commence the workshop at the start of the study and will be asked to attend one of three identical workshops in September 2018. Group B Participants will be the Control Arm.

Workshop

The workshop will consist of the 2-hour Stress Management and Resiliency Training (SMART) program developed by the Mayo Clinic. Participants will be asked to attend the workshop only once and will be asked to attend for the full duration. The learning objectives of the workshop are: (1) learn the neuroscience and behavioural aspects of human experience, particularly with respect to stress, resiliency, performance and wellness and (2) learn practical approaches to enhance engagement and emotional intelligence and thereby decrease stress and anxiety, increase resilience, enhance performance, and improve relationships. The outline course plan for the workshop is:

|  | Insight | Practices |
| --- | --- | --- |
| Module 1: Gratitude | Neuroscience of focus, fatigue and fear | Morning Gratitude, Gratitude Jar |
| Module 2: Mindful Presence | Attention Focus | Two-Minute Rule, Curious Moments |
| Module 3: Kindness | Neuroscience of fear | Kind Attention, Meditation |
| Module 4: Resilience Mindset | Insight on thinking | Resilient mindset with five principles: Gratitude, Compassion, Acceptance, Meaning, Forgiveness |

Questionnaires

Group A (Active Arm) participants will be asked to complete questionnaires prior to attending the 2-hour workshop at the time of enrolling in the study. They will also be asked to complete questionnaires following the workshop on 3 occasions over a 24-week follow up period (weeks 4, 12 and 24 following the workshop). Group B (Control Arm) participants will also be asked to complete the same questionnaires at the same follow up times as Group A. All requests to complete questionnaires will be made via email with one reminder 5-days later to those who have not replied.

The questionnaires are as follows:

1. ***Resiliency*.**

The Connor-Davidson Resilience Scale (CD-RISC) is a 25-item scale, which will be used to measure resiliency^1^. CD-RISC has been evaluated and has had good internal consistency (Cronbach’s α = .89), test-retest reliability (intra-class correlation coefficient = 0.87), and has positive correlation with multiple related measures with ability to distinguish between participants with lesser and greater resilience^15^.

1. ***Perceived stress*.** The Perceived Stress Scale (PSS) is a 10-item scale that provides a global measure of perceived stress^16,17^. Responses range on a 5-point scale from “never” to “very often.” A higher score indicates greater stress. The PSS correlates well with life events, stress measures, and social anxiety and has good internal consistency (Cronbach’s α = .87, .89, .91) in 3 different samples^18^.
2. ***Anxiety***. The Generalized Anxiety Disorder (GAD-7) scale is a 7-item questionnaire that asks how often, during the last 2 weeks, the participant was bothered by each symptom^19^. Response options are “not at all,” “several days,” “more than half the days,” and “nearly every day,” scored as 0, 1, 2, and 3, respectively. The GAD-7 has been found to have excellent internal consistency (Cronbach’s α = .92) and good test-retest reliability (intraclass correlation = 0.83).
3. ***Happiness.*** The Subjective Happiness Scale (SHS) is a 4-item measure of global subjective happiness where individuals can make an overall judgment about how happy (or unhappy) they are using a 7-point linear analogue scale^20^. The SHS has good internal consistency (Cronbach’s α = 0.86).

Completion of the questionnaires is expected to take approximately 10 minutes.

All participants will be assigned a unique study ID number that will be assigned to their questionnaires. The Master List (i.e., the link between the study ID and participants’ name and contact information) will be stored securely and separate from other study records, and will not leave the site. All study related documents will be stored in either locked offices and/or on password protected institutional computers.

E-learning support

Following completion of the 2-hour workshop, Group A (Active Arm) Participants will be enrolled in an online e-learning support program on a website developed by the Mayo Clinic. The aim of this is to support and reinforce the messages and techniques delivered in the 2-hour workshop. This will be accessible via a personal smartphone/iPad/PC/Mac. The online e-learning support program will last for 24-weeks in total, however since the participants were initially told that the program would only last 12 weeks they will be given the option of completing either the original 12 weeks or continuing for the full 24. The total time required using the e-learning support program for each participant over the 24-weeks is as follows:

- - Weeks 0-4: 45 minutes per week (broken up into e.g. short videos of 2-3 minutes)
  - Weeks 5-24: 10 minutes per week
- All Group A participants will be given a login to access the e-learning support website.
- Communication through the website will be only one-way (i.e. participants will be asked to read and watch educational materials only)
- The server/data is owned by the Mayo Clinic.
- The data entered in the e-learning course will be confidentially stored on a database within the Mayo Clinic IT Department.
- For the duration of the study, only the study team will access to the data
- The website will only collect information regarding the sessions the participants complete.
- All information entered in the e-learning website will be kept confidential.

Focus groups

Group A (Active Arm) Participants will be invited to join a focus group with other workshop participants 12-weeks after the workshop has been run. A choice of three dates will be offered. The key themes which will be explored during the focus group are as follows:

- How was the experience of participating in the workshop?
- Do you feel you have a better understanding of stress and resilience?
- Do you feel any different to your wellness since attending the workshop?
- What impact has it had both within and outside work settings?
- Have you or others noticed that any behaviours have changed?
- Have you or others noticed that any attitudes changed?
- Have you noticed any changes to any of your relationships (professionally or personally)?

Focus group interviews will be audio-recorded and transcribed verbatim. The audio recordings will be recorded on a non-encrypted device using a study specific audio recorder; they will then be transferred to a secure password protected TOH server immediately after the interviews, and deleted from the audio recording device immediately after being transferred to the secure server.

All study related documents will be stored in either locked offices and/or on password protected institutional computers.

The transcriptions will be de-identified, with all personal identifying information removed. Audio recording will be transcribed offsite. Personal identifiable information will not be discussed during the interview. Audio recordings will be sent to a professional transcriptionist trained in medical/medical education terminology at Verbatim Ottawa Services Inc. All audio recordings will be securely sent electronically via a password protected server. A copy of the audio file will be made before sending it offsite. The audio file will be kept on the secure password protected TOH server.

**Inclusion/Exclusion Criteria**

All full-time academic physicians in the Department of Medicine at the Ottawa Hospital/University of Ottawa will be invited to participate in the study.

**Risks/Benefits**

All phases of this study are low-risk. There is no physical risk to completing the questionnaires, workshop, e-learning or focus group. To avoid any risk associated with participating in a group workshop and focus group interview, all participants will be informed to not share personal information about other participants outside of the group. Participation will in no way affect participants’ current or future standing at the Ottawa Hospital or the University of Ottawa.

Assigning a unique study ID lowers the risk that the content from the study can be linked back to a specific participant. Participants may choose not to answer any question they do not like.

Participants may benefit by better understanding their attitudes toward physician wellness and further developing their resilience building skills. Participants may indirectly benefit from the researchers’ better understanding of how physician wellness and resilience can be taught in the future.

**Analysis of Results**

Quantitative (Questionnaires). For each measurement scale, the change from baseline will be compared between groups (Active Arm and d Control Arm) using the two-sample t-test. To supplement these analyses, the within-group change (baseline vs week 4/12/24) will be assessed for the Active Arm using the paired t-test. A sample size of 40 was selected for this study after weighing statistical considerations along with logistical and resource constraints. In general, for a

continuous outcome variable, a sample size of 40 provides statistical power (two-tailed, alpha=0.05) of >85% to detect a difference of 1 standard deviation between groups.

Qualitative (Focus Groups)

Constructivist grounded theory^21^ will inform the iterative data collection and analysis process. Transcripts will be analysed using a three-staged process of initial, focused, and theoretical coding. Themes will be identified using constant comparative analysis and grouped to look at the interrelationship of categories. Using a constructivist lens, the research team hopes to better understand how a community of practice for physician wellness can be developed and how this might increase knowledge and understanding of the process through which behaviour (individual resilience) and culture (social resilience) change occurs within an academic healthcare organization.

**Appendix A: Study Protocol**

**Recruit 40 Physicians**

**Randomize into 2 groups of 20**

**Group A (20 Physicians) Group B (20 Physicians)**

**Active Arm Control Arm**

| **Week** | **Group A** | **Group B** |
| --- | --- | --- |
|  | **Complete consent and questionnaires** | **Complete consent and questionnaires** |
| 0 | Attend 2-hour SMART training workshop  Enrol SMART online support |  |
|  |  |  |
| 4 | Complete questionnaires | Complete questionnaires |
|  |  |  |
| 12 | Complete questionnaires  End SMART online support (12 weeks) | Complete questionnaires |
|  |  |  |
| 16 | Attend 1-hour Focus Group |  |
|  |  |  |
| 24 | Complete questionnaires  End SMART online support (24 weeks – optional)  End study | Complete questionnaires  End Study |
|  |  |  |

**APPENDIX B: References**

1. Connor KM, Davidson JRT. Development of a new resilience scale: The Connor-Davidson Resilience Scale (CD-RISC). *Depress Anxiety*. 2003;18(2):76-82.

2. Wu G, Feder A, Cohen H, et al. Understanding resilience. *Front Behav Neurosci*. 2013;7.

3. Howe A, Smajdor A, Stöckl A. Towards an understanding of resilience and its relevance to medical training: Resilience and its relevance to medical training. *Med Educ*. 2012;46(4):349-356.

4. Adger WN. Social and ecological resilience: are they related? *Prog Hum Geogr*. 2000;24(3):347-364.

5. Wenger E. *Communities of Practice: Learning, Meaning, and Identity*. Cambridge University Press; 1998.

6. Lave J, Wenger E. Legitimate peripheral participation in communities of practice. In: Harrison R, Reeve F, Hanson A, Clarke J, eds. *Supporting Lifelong Learning: Perspective on Learning.* London: Routledge and Open University Press.; 2002:111-126.

7. Spilg E, Siebert S, Martin G. A social learning perspective on the development of doctors in the UK National Health Service. *Soc Sci Med*. 2012;75(9):1617–1624.

8. Mak WWS, Ng ISW, Wong CCY. Resilience: Enhancing well-being through the positive cognitive triad. *J Couns Psychol*. 2011;58(4):610-617.

9. Mealer M, Jones J, Newman J, McFann KK, Rothbaum B, Moss M. The presence of resilience is associated with a healthier psychological profile in intensive care unit (ICU) nurses: Results of a national survey. *Int J Nurs Stud*. 2012;49(3):292-299.

10. McGarry S, Girdler S, McDonald A, et al. Paediatric health-care professionals: Relationships between psychological distress, resilience and coping skills: Psychological distress and coping skills. *J Paediatr Child Health*. 2013;49(9):725-732.

11. Lü W, Wang Z, Liu Y, Zhang H. Resilience as a mediator between extraversion, neuroticism and happiness, PA and NA. *Personal Individ Differ*. 2014;63:128-133.

12. Fox S, Lydon S, Byrne D, Madden C, Connolly F, O’Connor P. A systematic review of interventions to foster physician resilience. *Postgrad Med J*. 2017;94(1109):162-170.

13. Sood A, Prasad K, Schroeder D, Varkey P. Stress Management and Resilience Training Among Department of Medicine Faculty: A Pilot Randomized Clinical Trial. *J Gen Intern Med*. 2011;26(8):858-861.

14. Sood A, Sharma V, Schroeder DR, Gorman B. Stress Management and Resiliency Training (SMART) Program among Department of Radiology Faculty: A Pilot Randomized Clinical Trial. *EXPLORE J Sci Heal*. 2014;10(6):358-363.

15. Campbell‐Sills L, Stein MB. Psychometric analysis and refinement of the connor–davidson resilience scale (CD‐RISC): Validation of a 10‐item measure of resilience. *J Trauma Stress*. 2007;20(6):1019-1028.

16. Cohen S, Kamarck T, Mermelstein R. A Global Measure of Perceived Stress. *J Health Soc Behav*. 1983;24(4):385-396.

17. Cohen S, Williamson GM. Perceived Stress in a Probability Sample of the United States. In: Spacapan S, Oskamp S, eds. *The Social Psychology of Health*. Claremont Symposium on Applied Social Psychology. Newbury Park, Calif. Sage Publications; 1988:31-67.

18. Lee E-H. Review of the Psychometric Evidence of the Perceived Stress Scale. *Asian Nurs Res*. 2012;6(4):121-127.

19. Spitzer RL, Kroenke K, Williams JB, Löwe B. A brief measure for assessing generalized anxiety disorder: the GAD-7. *Arch Intern Med*. 2006;166(10):1092–1097.

20. Lyubomirsky S, Lepper HS. A Measure of Subjective Happiness: Preliminary Reliability and Construct Validation. *Soc Indic Res*. 1999;46(2):137-155.

21. Charmaz K. *Constructing Grounded Theory*. Sage; 2014. https://us.sagepub.com/en-us/nam/constructing-grounded-theory/book235960. Accessed December 21, 2017.
